# A prospective multicenter clinical feasibility study of a new automatic speaking valve for postlaryngectomy voice rehabilitation

**DOI:** 10.1007/s00405-016-4304-y

**Published:** 2016-09-14

**Authors:** L. Lansaat, B. J. de Kleijn, F. J. M. Hilgers, B. F. A. M. van der Laan, M. W. M. van den Brekel

**Affiliations:** 1 Department of Head and Neck Oncology and Surgery, Netherlands Cancer Institute, Plesmanlaan 121, 1066 CX Amsterdam, The Netherlands; 2 Department of Otorhinolaryngology-Head and Neck Surgery, University of Groningen, University Medical Center Groningen, Groningen, The Netherlands; 3 Institute of Phonetic Sciences (ACLC), University of Amsterdam, Amsterdam, The Netherlands; 4 Department of Maxillofacial Surgery, Academic Medical Center, University of Amsterdam, Amsterdam, The Netherlands

**Keywords:** Total laryngectomy, Automatic speaking valve, Heat and moisture exchanger, Compliance, Voice

## Abstract

Evaluation of short- and long-term clinical feasibility and exploration of limitations and advantages of a new automatic speaking valve (ASV) for laryngectomized patients with integrated HME, the Provox FreeHands FlexiVoice (FlexiVoice). This ASV not only enables automatic, but also manual closure of the valve. A multicenter, prospective clinical study in 40 laryngectomized patients was conducted. Participants were asked to use the FlexiVoice for 26 weeks. The primary outcome measure was long-term compliance. Secondary outcome measures were: patient preference, hours of FlexiVoice use, device life of adhesive, voice and speech quality, and quality of life. After 26 weeks, 15 patients (37.5 %) were using the FlexiVoice on a daily basis, for a mean of 12.64 h/day (SD ± 5.03). Ten patients (25 %) were using the device on a non-daily basis, for a mean of 3.76 h/day (SD ± 2.07). The remaining 15 patients (37.5 %) discontinued using the FlexiVoice. Sixty percent of the 25 long-term users applied both automatic and manual closure of the valve. Unpredictable fixation of the adhesive was the main reason for discontinuing or not using the FlexiVoice on a daily basis. Overall, 18 patients (45 %) preferred the FlexiVoice, 16 patients (40 %) their usual HME, 3 patients (7.5 %) their usual ASV, 1 patient (2.5 %) preferred no device at all, and in 2 patients preference was not recorded. The minor technical issues identified could be corrected. The Provox FreeHands FlexiVoice appears to be a useful ASV, which allows for hands-free speech in a larger proportion of laryngectomized patients in the present cohort. The additional manual closure option of the device is beneficial for maintaining the adhesive seal longer.

## Introduction

Total laryngectomy (TL) results in significant anatomical changes. The alimentary and respiratory tracts are separated and a permanent stoma is created in the neck [1]. To compensate for the loss of the voice box, currently primary insertion of a tracheoesophageal voice prosthesis is the gold standard for restoring pulmonary-driven speech [2]. To compensate for the functional loss of the upper respiratory tract and to prevent and/or treat pulmonary problems, such as excessive coughing and mucus production, continuous use of heat and moisture exchanger (HME) has proven to be effective [3–5]. Speaking with a voice prosthesis requires airtight occlusion of the stoma with a finger to divert the pulmonary air into the pharyngoesophageal segment or neoglottis, where mucosal vibrations produce the sound for speech. Airtight stoma occlusion has become easier after the development of specialized HMEs, which improve maximum phonation time and dynamic loudness range and thus compliance rate [6]. However, with these HMEs, it is still necessary to use a finger to occlude the stoma for speech production. To overcome this drawback of tracheoesophageal speech and to obtain hands-free speech, automatic speaking valves (ASVs) have been developed. These devices contain a flexible membrane that stays open during normal calm breathing, but closes through the natural increase in air pressure when speaking is initiated [7, 8]. Several ASVs are presently available. The first were the Blom Singer and Bivona tracheostoma valves in the eighties and nineties of the last century [8–10]. Later, several other valves became available, such as the Eska-Herrmann and ADEVA valves [11, 12]. In 2003, the Provox FreeHands HME (further called FreeHands; Atos Medical, Hörby, Sweden) was introduced, which was the first automatic speaking valve with an integrated HME for simultaneous pulmonary rehabilitation [7]. In a long-term (6 months) study, the success rate (defined as patients using this ASV on a daily basis) was 19 % [13]. Additionally, 57 % of patients in this study used the device on a non-daily basis at special occasions, such as during shopping or social activities [13]. The main reason for not using the FreeHands on a daily basis was the unpredictable fixation of the adhesive to the peristomal skin. This is the main drawback for all ASVs. For a considerable number of patients, it can be problematic to obtain a good and long-lasting seal of the adhesive to withstand the pressure necessary for speaking [14–17].

To further improve patient friendliness and compliance of automatic speech, a new automatic speaking valve was developed, the Provox FreeHands FlexiVoice (further called FlexiVoice; Atos Medical AB, Hörby, Sweden). This new ASV contains a renewed mechanism to lock and unlock the speaking membrane. The air pressure needed to close the membrane is lower than in the earlier FreeHands device, because the available membranes are more flexible. Moreover, there is a novel option to alternatively occlude the device manually: a front opening also allows speech through finger occlusion of the device, even when the membrane is locked, e.g., during physical exertion. Lastly, the coughing mechanism is adapted, which also allows easy repositioning of the valve after coughing.

The objective of this prospective clinical study is to evaluate the short- and long-term feasibility of the FlexiVoice, in combination with the currently available attachments, and to explore its limitations and advantages.

## Methods

The study was carried out at two tertiary care cancer centers. Inclusion criteria were: TL, 18 years or older, use of an HME and/or ASV, use of a voice prosthesis irrespective of the voice quality, minimum of 3 months after TL and/or postoperative (chemo-) radiotherapy. Exclusion criteria were: inability to remove or operate the FlexiVoice, active recurrent or metastatic disease, inability to understand the patient information, to give informed consent, and/or to complete diaries. The study was performed according to the protocol approved by the institutional review boards and all patients were enrolled in the study between May 2014 and August 2014. Signed informed consent was obtained from all participants.

**Fig. 1:**
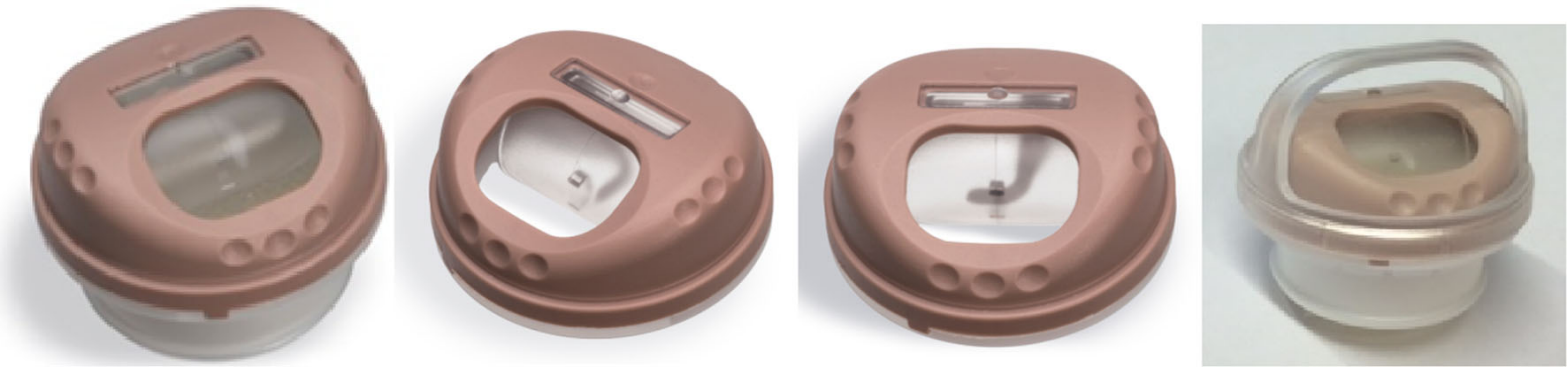
*Left* Provox FreeHands FlexiVoice. The heat and moisture exchanger (HME) is attached and the flexible membrane is closed. *Middle left* ‘automatic speaking mode’. *Middle right* ‘locked mode’: the patient can rotate the top of the device and the membrane is locked by a hook that grabs a ring at the backside of the membrane. *Right* the arch is attached. It prevents the front opening being occluded by clothing (*left 3 pictures* by courtesy of Atos Medical)

The FlexiVoice is shown in Fig. 1 (left). It combines pulmonary rehabilitation using an HME, with voice rehabilitation using an ASV, which also facilitates manual occlusion. The device is attached in front of the stoma of a laryngectomized patient, who is using a voice prosthesis for speech. There are different attachment options for the subjects to choose from (various stoma adhesives, laryngectomy tubes and buttons). The base of the device is the HME cassette and the speaking valve is anchored on top of that HME cassette. The speaking valve has a front opening and an internal flexible membrane. When the patient starts to speak, the natural increase in exhalation airflow closes the membrane. The exhaled air is thus diverted through the voice prosthesis, which allows hands-free tracheoesophageal speech. Alternatively, the patient can choose to occlude the opening in the front with his/her finger to speak. Rotating the top of the device moves the FlexiVoice into the ‘locked mode’, or into the ‘automatic speaking mode’ (Fig. 1, middle left). In ‘locked mode’, the membrane is prevented from closing with a hook grabbing a ring at the backside of the membrane (Fig. 1, middle right). Thereby, the patient is ensured of unrestricted and comfortable breathing during physical exertion, still allowing manual occlusion for speech. There are three versions of the speaking valve, each with a different flexibility/strength of the membrane: light, medium and strong. When coughing is needed, the membrane pops out through the front opening and the patient can push the membrane back manually. There is an optional arch that can be attached on top of the device to prevent the front opening of being occluded by clothing (Fig. 1, right).

After inclusion, patients used the FlexiVoice for the duration of a maximum of 6 months. The primary objective was to assess long-term compliance, based on various aspects of the ASV addressed in study-specific questionnaires. Secondary outcome measures were: patient preference, hours of FlexiVoice use, device life of adhesive, voice and speech quality and quality of life. The questionnaires were completed at the time of inclusion, after 4 weeks and after 26 weeks.

The study specific questionnaires addressed the use of adhesive, effort needed to speak, noises produced by the FlexiVoice, coughing mechanism, appearance, functioning of the membrane, use of the ‘locked mode’/‘automatic speaking mode’, manual occlusion, device life of adhesive, voice quality, speech quality and intelligibility. Additionally, patients rated satisfaction regarding the FlexiVoice, their usual ASV/HME (if applicable), the device life of their adhesive, and their voice quality on a 10-cm Numeric Rating Scale (NRS) (0 = worst and 10 = best).

**Fig. 2:**
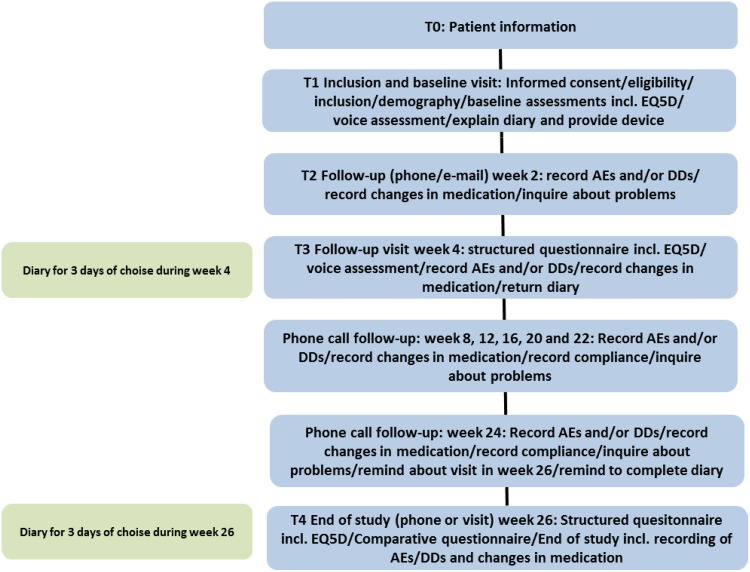
Study flowchart

Quality of life was assessed using the EuroQOL-5 Dimension-5 Level questionnaire (EQ5D5L). This instrument is validated using scores in five health-care dimensions (mobility, self-care, daily activities, pain/discomfort and anguish/depression) and a 100-mm VAS [18]. Voice and speech quality assessment consisted of reading a text, numbering breathing pauses, maximum phonation time (vowel /a/ and counting) and dynamic loudness range (with calibrated decibel meter). During the study period, patients kept a diary twice for 3 days in the week before each follow-up visit to record daily hours of FlexiVoice use. At the end of the study, patients were asked to complete comparative questionnaires. Patients were asked to compare the FlexiVoice with the usual ASV and/or HME and to answer questions regarding preference and future use. Patients were contacted by telephone 2 weeks after inclusion and at monthly intervals until 26 weeks of follow-up. If needed, additional practical support from the speech pathologist or the study coordinator was offered. Figure 2 provides an overview of the study design.

### Statistics

As this was deemed to be an uncomplicated feasibility study in patients familiar with the use of peristomal adhesives and HME devices and no risks associated with participation in the study were expected, the dropout rate was estimated to be <5 %. Statistical analyses were conducted using IBM^®^ SPSS^®^ 22.0. Frequencies were explored using the Kolmogorov–Smirnov test. Parametrically distributed data are shown as mean ± standard deviation and analyzed using the paired *T* test. Non-parametric data are presented as median (interquartile range) and were analyzed using the Wilcoxon signed rank test. The Likert scales rendered ordinal data from three related samples. This data was analyzed using the Friedman test. If the groups differed significantly, a Wilcoxon signed rank test was used to determine which groups were different. A *p* value <0.05 is considered to be significant.

## Results

In total, 41 laryngectomized patients were entered in the study, 21 in one institute and 20 in the other. One patient subsequently had to be excluded from the study and further analysis, because the language barrier was larger than anticipated and he did not understand the patient information. Thus, the remaining 40 patients, 36 males and 4 females, were included for analysis. Patient demographics and clinical information are provided in Table 1. At baseline, 27 patients were not using an ASV (67.5 %), 12 patients were using an ASV in combination with an HME and (30 %) 1 patient was using only an ASV (2.5 %), also during the night (all ASVs were the FreeHands [7]). Of those 13 ASV users (32.5 %), 8 patients were using the ASV on a daily basis (20 %) and 5 patients on a non-daily basis (12.5 %). The mean ASV use of the eight daily users was 13.25 h and of the five non-daily users 3.26 h. Of the 27 non-users, 19 (70 % did have experience with an ASV before entering the study and 6 (15 %) did not (data in 2 patients was missing). Most ASV users were using one of the ‘stronger’ adhesives, such as the Provox StabiliBase adhesive (Atos Medical AB, Hörby, Sweden). The self-reported median device life of the adhesive was 19 h (range 1–168) when using an ASV (*n* = 12; data in 1 patient was missing). Patients’ satisfaction regarding adhesive device life when using the ASV was rated 7.16 on a scale 1–10 (NRS; SD ± 2.35; *n* = 11). This information was missing in two patients. For the non-ASV users, the median device life of the adhesive was 24 h (range 6–168 h; *n* = 26, data missing in 1 patient).

**Table 1:** Patient characteristics

Characteristics	Value	%
Gender		
Male	36	90.0
Female	4	10.0
Age at TL	Mean 56.3 years (SD ± 9.4)	
Age at entry	Median 63.5 years (SD ± 8.91)	
Post-TL	Median 74.5 months (range 3–317 months)	
TL		
Standard	32	80.0
+Reconstruction	6	15.0
Gastric pull-up	1	2.5
Information missing	1	2.5
Radiotherapy		
No	1	2.5
Preoperative	30	75.0
Postoperative	9	22.5
ASV use		
No	27	67.5
Only ASV	1	2.0
ASV + HME	12	30.0
Experience with ASV		
No	6	15.0
Yes	32	80.0
Information missing	2	5.0

*TL* total laryngectomy, *ASV* automatic speaking valve, *HME* heat and moisture exchanger

### Assessment at 4 weeks

At the 4-week follow-up, 36 patients were still in the study and 4 had stopped using the FlexiVoice. Nineteen of the original 40 patients (47.5 %) used the FlexiVoice on a daily basis, for a mean of 10.87 h/day (SD ± 4.67; *n* = 18; missing data in 1). Seventeen of the original 40 patients (42.5 %) used the FlexiVoice on a non-daily basis, for a mean of 6.82 h/day (SD ± 6.12; missing data *n* = 1). The reasons for not using the FlexiVoice on a daily basis are shown in Table 2. Most common were unpredictable fixation of the peristomal adhesive (*n* = 3) and familiarity of the usual HME/ASV (*n* = 3). Furthermore, for the four patients, who discontinued between inclusion and the 4-week follow-up, the reasons given are also summarized in Table 2.

**Table 2:** Reasons for discontinuing the study and not using FlexiVoice on a daily basis, and occasions when using FlexiVoice in the latter non-daily user group

Reasons for discontinuing the study between inclusion and 4 weeks^a^ Unpredictable adhesion of adhesive (*n* = 1); excessive mucus (already at baseline; *n* = 1); voice prosthesis problem (*n* = 1); recurrent disease (*n* = 1)
Reasons for not using FlexiVoice on a daily basis at 4 weeks^a^ Unpredictable adhesion of adhesive (*n* = 3); familiarity with usual HME/ASV (*n* = 3); less easy voicing (*n* = 3); “FlexiVoice cannot be used without HME” (*n* = 2); skin irritation with adhesive (*n* = 1); uncomfortable breathing resistance (*n* = 1); more mucus (*n* = 1); problem with voice prosthesis (*n* = 1); high T-shirt difficulty (*n* = 1); mostly using esophageal speech (*n* = 1); air leakage with manual occlusion (*n* = 1); unintentional closing membrane (*n* = 1); when home alone, ASV not necessary (*n* = 1)
Reasons for discontinuing the study between 4 and 26 weeks^a^ Unpredictable adhesion of adhesive (*n* = 6); too high breathing resistance (*n* = 6); soft voice (*n* = 2); too easy closing membrane (*n* = 2); usual ASV easier (*n* = 2); not easy with certain clothes (*n* = 1); too much speaking effort (*n* = 1); annoying sounds (*n* = 1); excessive mucus (already at baseline; *n* = 1); poor intelligibility (*n* = 1)
Reasons for not using the FlexiVoice on a daily basis at 26 weeks^a^ Unpredictable adhesion of adhesive (*n* = 4); more mucus (*n* = 2); uncomfortable breathing resistance (*n* = 2); soft voice (*n* = 2); preference for usual HME (*n* = 2); less easy voicing (*n* = 1); when home alone ASV not necessary (*n* = 1); too fast popping out membrane (*n* = 1); too loose arch (*n* = 1)
Occasions when using FlexiVoice in the non-daily user group at 26 weeks^a^ At home (*n* = 9); during social activities (*n* = 6); in special situations [e.g., when driving a car, on a quiet day, only during patient counseling (e.g., one of the less then once a month patients) (*n* = 3)]; during the whole day (*n* = 2); at the work place (*n* = 1)

*HME* heat and moisture exchanger, *ASV* automatic speaking valve

^a^ More options per patient possible

### Assessment at 26 weeks

At 26 weeks, 25 patients still used the FlexiVoice, whereas the remaining 11 patients had discontinued its use. Fifteen of these 25 patients (37.5 % of the original 40 patients) used the FlexiVoice on a daily basis, for a mean of 12.64 h/day (SD ± 5.03; *n* = 14; missing data *n* = 1). Ten patients (25 % of the original 40 patients) used the device on a non-daily basis, for a mean of 3.76 h/day (SD ± 2.07; *n* = 6; missing data *n* = 4). The type of surgery (standard TL versus pharyngeal reconstruction) did not influence ASV use. Unpredictable fixation of the adhesive was the main reason (*n* = 4) for not using the FlexiVoice on a daily basis at 26 weeks follow-up. All reasons are shown in Table 2, as well as the reasons for discontinuing between 4 and 26 weeks. Actual FlexiVoice use in the ten non-daily users was: 5–6 days/week (*n* = 1), 3–4 days/week (*n* = 4), 1–2 days/week (*n* = 2), 1–2 days/month (*n* = 1) and less than once per month (*n* = 2). Occasions when using the FlexiVoice in this non-daily user group are also given in Table 2.

**Fig. 3:**
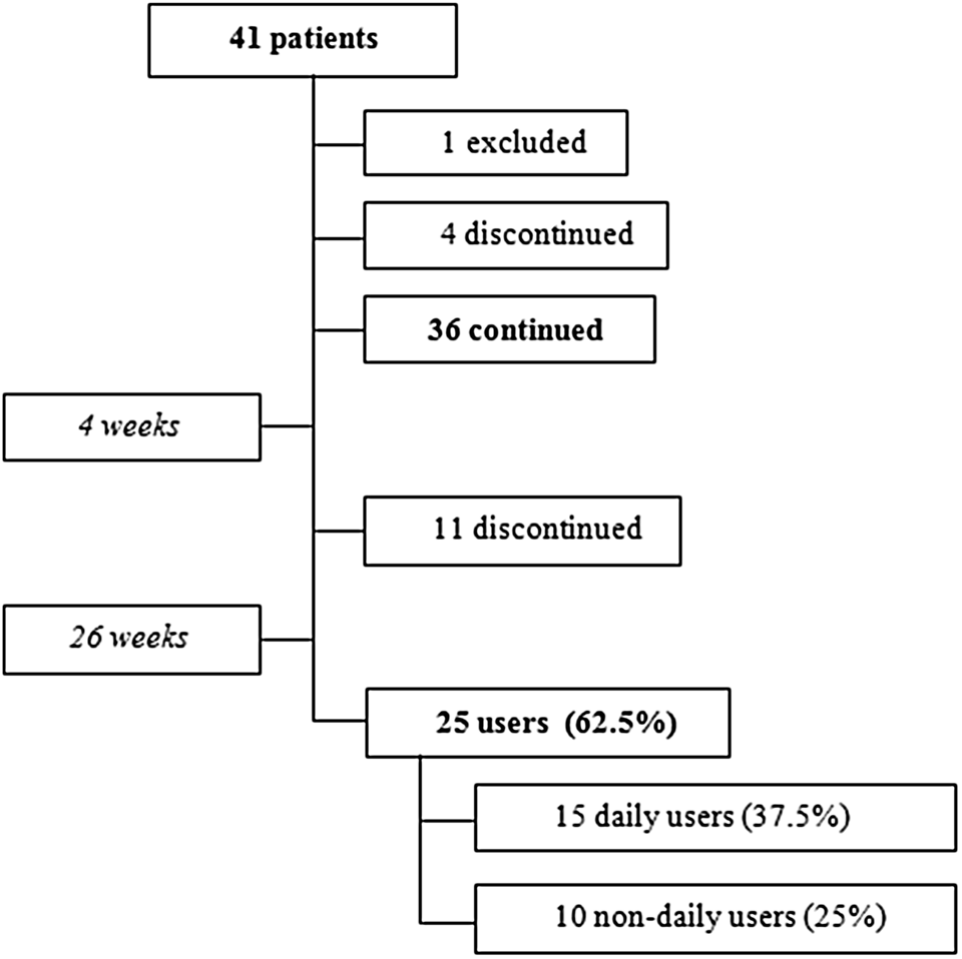
Flowchart of patient compliance

**Fig. 4:**
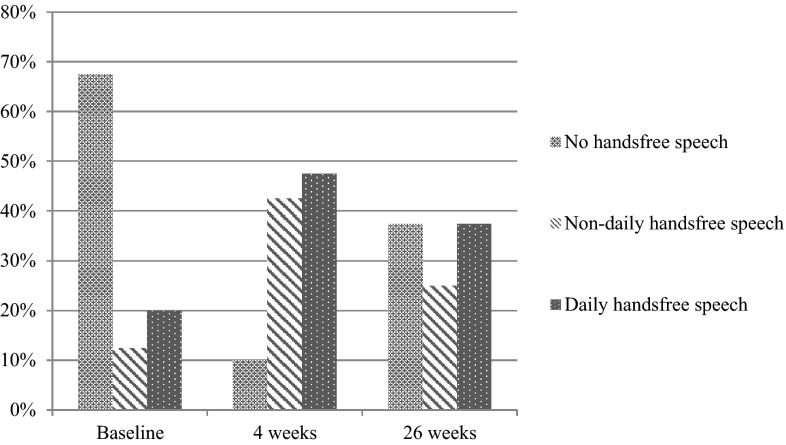
Compliance rates regarding hands-free speech (*n* = 40)

Thus, in total, 15 patients decided to end the study earlier than planned, of whom 2 patients did use an ASV at baseline (and went back to that) and 13 patients did not use an ASV at baseline. An overview of patient numbers, compliance and rates regarding hands-free speech at different moments in the study is given in Figs. 3 and 4.

With respect to the attachment of the FlexiVoice to the stoma at 26 weeks, of the 25 FlexiVoice users 13 were using the StabiliBase adhesive to attach the FlexiVoice, 4 FlexiDerm, 3 OptiDerm, 3 StabiliBase OptiDerm, 1 Regular, 1 XtraBase, 3 LaryTube and 2 LaryButton (more options per patient possible; all adhesives/devices are from Atos Medical AB, Hörby, Sweden). The self-reported median daily device life of the adhesive was 8 h (range 0.25–168), when using the FlexiVoice (*n* = 23; 2 patients were not using an adhesive, but a laryngectomy button). Patients’ satisfaction regarding adhesive device life with the FlexiVoice was rated on average 6.46 (NRS; SD 2.61; *n* = 23). Four of 11 patients (36 %), who used an ASV at baseline, changed their choice of adhesive(s), and 8 of 14 patients (57 %), who did not use an ASV at baseline, also changed their choice of adhesive(s).

With regard to the practical aspects of the FlexiVoice, patients were, e.g., asked to indicate if the membrane was popping out while coughing. Almost all patients answered affirmative and all patients found it easy to push the membrane back. When asked if the membrane sometimes closed unintentionally, 12 patients answered in the affirmative and 13 patients answered as negative. This happened mostly when patients were physically active (*n* = 11). Seventeen of 25 patients (68 %) did use the ‘locked mode’ with a median of 1.5 times per day (range 0–10). All patients used automatic occlusion and 15 of 25 long-term users (60 %) used both automatic occlusion and manual occlusion. The main reasons for using manual occlusion were: loosening of the adhesive makes hands-free speech impossible, but still allows speech with manual occlusion (*n* = 8), and the voice is louder (*n* = 3). Seventeen of 25 patients indicated good intelligibility when using the FlexiVoice in automatic speaking mode, 2 found the intelligibility reasonable, 4 moderate and 2 poor. No significant differences in quality of life (according to the EQ5D5L) were found between baseline, at 4 weeks and at 26 weeks (data not shown). There were also no significant differences of the objective voice parameters assessed between baseline and 26 weeks follow-up (see Table 3).

**Table 3:** Objective voice assessment: hands-free speech parameters at baseline and 26 weeks [median (range)]

	Baseline (*n* = 13)	26 weeks (*n* = 23*)
Breathing pauses (*n*)	23 (16–68)	24 (9–66)
Total length of text (min)	1:19 (1.05–1.58)	1:14 (0.56–2.37)
Max phonation time (s)		
Prolonged /a/	7.30 (2.70–30.40)	7.58 (2.57–32.35)
Counting	11.1 (3.90–19.10)	11.76 (2.50–45.00)
Dynamic loudness range (dB)		
Softest	58 (42–70)	58 (51–69)
Comfortable	67.3 (62–74)	66 (55–77)
Loudest	77 (73–84)	79 (70–92)

There are no significant differences between the baseline and 26 weeks

* Two patients did not complete the voice assessment or not all items, because one could not read and the other could not read Dutch, and his adhesive did not last long

### Comparison with usual ASV

At 26 weeks, 11 patients did compare the FlexiVoice with their usual ASV (in all patients, the FreeHands). Regarding the coughing mechanism, six patients preferred the coughing mechanism of the FlexiVoice and five expressed no preference. Regarding the overall voice quality, five patients preferred the FlexiVoice, five had no preference and one preferred the FreeHands. Regarding speaking effort, five patients preferred the FlexiVoice and six expressed no preference. Membrane-closing noise was reportedly less with the FlexiVoice in four patients, with the FreeHands also in four and similar in three patients. Furthermore, 4 of these 11 ASV patients reported that they could speak longer on one intake of breath with the FlexiVoice, whereas 7 patients expressed no difference in this respect. Regarding appearance, eight patients preferred the FlexiVoice and three had no cosmetic preference. Overall, one of these 11 patients preferred to stay with his original ASV.

**Fig. 5:**
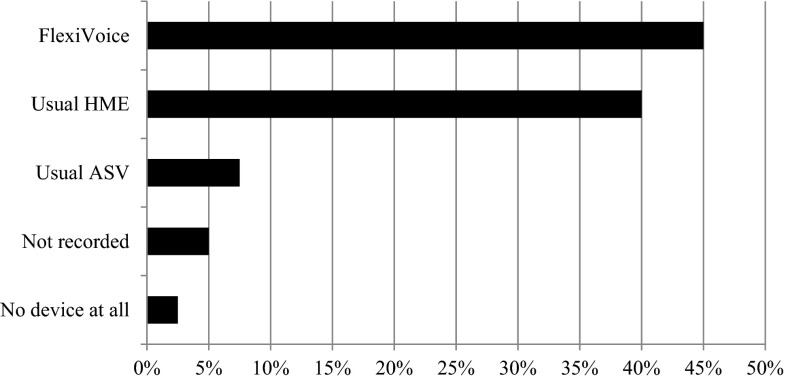
Preference after 26 weeks (*n* = 40). *ASV* automatic speaking valve, *HME* heat and moisture exchanger

With regard to overall stoma occlusion preference at 26 weeks, 18 patients preferred the FlexiVoice (45 %), 16 (40 %) their usual HME, 3 (7.5 %) their usual ASV and 1 (2.5 %) no device at all. The preference in the two patients (5 %), who stopped before the 4 weeks assessment because of recurrent disease/voice prosthesis problem, was not recorded. Figure 5 shows the preferences. Finally, regarding future use, 16 out of 40 patients (40 %) would continue to use the FlexiVoice daily, 8 patients reported that they would use the FlexiVoice on a non-daily basis (20 %) and 16 patients would not continue with the FlexiVoice.

During this study, 17 clinical and device-related events were registered. One event concerned aspiration of the voice prosthesis, which was not FlexiVoice related (voice prosthesis was retrieved from the trachea; no further morbidity). There were 13 device-related events, most of which (*n* = 6) concerned the arch that fitted too loosely on the FlexiVoice. Based on these reports the arch underwent a redesign, which solved this issue. Another issue (*n* = 3) was air leakage from the device when closed manually, which was solved by adapting the attachment of the HME to the FlexiVoice. The other four concerned membrane issues, which also led to minor design changes solving this. The remaining three registered events concerned one patient, who complained twice about excessive moisture collection in the device, and one patient, who complained about excessive mucus production (already present at baseline).

## Discussion

This prospective clinical feasibility study on the evaluation of the Provox FreeHands FlexiVoice, a new ASV for laryngectomized patients using prosthetic voice, shows favorable results. The daily use of hands-free speech in this cohort increased from 20 % (8/40) at baseline to 37.5 % (15/40) at 26 weeks follow-up, with 10 of the original 13 FreeHands users switching to the new FlexiVoice. Moreover, besides the original five non-daily FreeHands users, there were five additional non-daily users for a total of ten patients (12.5 % at baseline compared to 25 % at 26 weeks), who used/converted to the new FlexiVoice device. Thus, for almost two-thirds of the patients, the FlexiVoice is a valuable option, whereas one-third of the patients remain fully dependent on finger occlusion. The expectation that the new features/adaptations of this new automatic speaking valve would result in an increased proportion of patients able to use hands-free speech seems to be met. An interesting observation is that the number of hours of ASV use was not different between both devices. Daily ASV users applied the device 13.25 h per day at baseline, 10.87 h at 4 weeks and 12.64 h at 26 weeks. For the non-daily users, these numbers were 3.26, 6.82 and 3.76 h, respectively. This is in line with the fact that daily users tend to apply the ASV only during daytime and non-daily users only at special occasions.

Several factors could have contributed to this increased hands-free speaking rate. At the end of the study, 60 % of the FlexiVoice users (15 out of 25 patients) used automatic occlusion in combination with manual occlusion and the main reason for switching to manual occlusion was the unpredictable fixation of the peristomal adhesive. The advantage of this new feature of the FlexiVoice is that, when the adhesive starts loosening, it is still possible to use the device by occluding the opening in the front with a finger, which maintains the seal somewhat longer, obviating the immediate need to switch back to a normal HME and/or change the adhesive. An effective coughing mechanism is another important aspect of hands-free speech, both for relieving the tracheal pressure and maintaining a good seal of the adhesive. In almost all patients, the membrane popped out on coughing and it was easy to push the membrane back; this might have been an additional reason for patients to continue using the FlexiVoice. It cannot be excluded, though, that an important reason for this increased use might have been that the StabiliBase and StabiliBase OptiDerm adhesives, with a more stable and more anatomically shaped conical base, were popular adhesives in this study population and that these were not yet available during previous studies evaluating hands-free speech [19]. Lastly, the increased number of patients using hands-free speech, in part, also could have been an effect of the renewed attention to an ASV some time later during the follow-up, something that should be kept in mind during regular aftercare of laryngectomized patients. A failure to acquire hands-free speech early on might still be correctable later.

There are several comparable studies on ASVs. The study of Op de Coul et al. (2005) evaluating the FreeHands device describes a higher overall compliance rate of 76 % than the 62.5 % (daily and non-daily users) in the present study [13]. However, the daily use of hands-free speech has doubled from 19 to 37.5 % in the present study, as has the number of h/day from a median of 5 h/day with FreeHands to more than 12 h/day with the FlexiVoice. In their study on the FreeHands device in 14 patients, Tervonen et al. found daily use in only 7 %, non-daily use in 86 % and non-use in 7 % [20]. These figures are again different from the ones found in the present study, but the numbers of patients in the Tervonen study are quite low, and there was a selection bias because only patients with a clear voice when using an HME were included [20]. In the present study, no such selection was made and also patients with less clear voices were represented. The heterogeneity of our patient sample (with 32 standard TLs, 6 pharynx reconstructions and 1 gastric pull-up) certainly results in a wide range of voice qualities, but this in fact did not influence long-term ASV use: reconstructed patients did as good as standard TL patients in this respect. Schwarz et al. described an acceptance rate of 62 % of patients using the device for at least 2 h per day during 4 weeks [21]. Such early results might not be that relevant, because in our study, compliance rate regarding daily use dropped from 47.5 % after 4 weeks to 37.5 % after 26 weeks, and the overall compliance dropped from 90 % at 4 weeks to 62.5 % at 26 weeks. To properly assess the compliance regarding a complicated device such as an ASV, a longer than a 4-week follow-up period is thus needed to provide relevant information. The study of Lorenz et al. on the FreeHands device in 24 patients does have a similar follow-up time as the present study (6 months), and the results are quite comparable with 42 % daily users and 29 % non-daily users [22]. However, the mean number of hours in the daily users, just like in the Op de Coul study, was also lower (8.4 h) than with the new FlexiVoice. Furthermore, the firsthand comparison of the FreeHands and FlexiVoice, possible in the present study for 11 patients, showed interesting differences, also supporting the assumption that the new design features of the FlexiVoice indeed improved its usability. The reported differences in favor of the FlexiVoice were less speaking effort, better overall voice quality, better appearance, easier and less noisy coughing mechanism and less noisy closing of the speaking membrane.

The key success factor of hands-free speech is maintaining the seal of the adhesive [7–9, 19, 21, 23]. It is important to realize that, as reported in the results, the median device life of the adhesive among ASV users at baseline was 19 h (range 1–168), whereas this was 8 h (range 0.25–168) reported in diaries after 26 weeks using the FlexiVoice. A possible explanation for this considerable difference in adhesive device life is that the patients, who used an ASV at baseline, were successful because of their excellent adhesive seal. Nevertheless, this study also shows once more that difficulties with adhesion of the adhesive to the skin are still a limiting factor, despite the easier closing of the more flexible/less strong membranes and the wider range of adhesives available for laryngectomized patients. More research and product development are thus needed to further improve peristomal attachment.

No significant differences in objective voice assessment were found between baseline, after 4 weeks and after 26 weeks, which shows that patients using the FlexiVoice are able to produce the same voice and speech quality compared to their baseline measurement with FreeHands as well as with HME. This is in contrast with the Op de Coul study, in which several voice parameters, such as maximum phonation time and dynamic loudness range, were significantly better when speaking with the HME [13]. The lack of such difference in the present cohort seems to further confirm the design improvements of the FlexiVoice.

The present study has some limitations. Although the only inclusion criterion was the ability to tolerate an HME, there still might have been a selection bias toward more motivated patients. Furthermore, some of the variables that (also) might influence hours of use of the FlexiVoice were not collected. In hindsight, it would have been interesting to not only let the patients report daily hours of FlexiVoice use in diaries, but also to ask the patients to give insight into the intensity of speech during the day. Also, information of stoma dimensions and local anatomy might have been of value to correlate duration of adhesive seal and thus hands-free speaking time [23]. Another limitation could be that for experienced ASV users, it is easier to handle a new ASV. However, they were willing to switch, only if the new device was really perceived as an improvement. Otherwise, they tended to stay with their original device. The fact that 10 of the original 13 ASV users did switch to the new ASV suggests that this limitation does not play a major role in this cohort.

In conclusion, the Provox FreeHands FlexiVoice is a useful ASV, which seems to allow for hands-free speech in a larger proportion of laryngectomized patients in the present cohort. The additional manual closure option of the FlexiVoice is experienced as beneficial for maintaining the adhesive seal longer.
